# Anatomical evaluation of the esophagus using computed tomography to predict acute gastroparesis following atrial fibrillation ablation

**DOI:** 10.1002/joa3.12625

**Published:** 2021-08-28

**Authors:** Daisuke Yakabe, Yusuke Fukuyama, Masahiro Araki, Toshihiro Nakamura

**Affiliations:** ^1^ Department of Cardiology Clinical Research Institute National Hospital Organization Kyushu Medical Center Fukuoka Japan

**Keywords:** atrial fibrillation, catheter ablation, complication, gastroparesis, vagal nerve injury

## Abstract

**Background:**

Catheter ablation for atrial fibrillation is an effective treatment; however, periesophageal vagal nerve injury is not rare and sometimes results in acute gastroparesis (AGP) after atrial fibrillation ablation (AFA). We sought to investigate the incidence and risk factors of AGP via preprocedural computed tomography (CT) analysis.

**Methods:**

We retrospectively reviewed 422 patients who underwent index AFA at our center. Using contrast‐enhanced CT performed before ablation, the anatomical characteristics of the esophagus were compared between patients with and without post‐ablation AGP. AGP was diagnosed by the presence of symptoms, fasting abdominal X‐ray radiography as a screening test, and additional abdominal imaging.

**Results:**

Of the 422 patients (age, 67 ± 11 years; male, 68.5%; cryoballoon, 63.7%), AGP developed in 14 (3.3%) patients, and six of 14 patients were asymptomatic. AGP resolved in all patients within 4 weeks without invasive treatment. In the AGP group, the esophagus was frequently located on the vertebra (middle‐positioned esophagus) (AGP vs non‐AGP, 42.9% vs 11.5%; *P* = .01), and additional posterior wall ablation was frequently performed (50.0% vs 14.5%; *P* = .02). In the multivariate analysis, middle‐positioned esophagus (*P* = .02; odds ratio, 9.0; 95% confidence interval [CI], 1.5‐53.3) and additional posterior wall ablation (*P* = .01; odds ratio, 7.6; 95% CI, 1.5‐42.1) were independent predictors of AGP.

**Conclusions:**

Anatomical evaluation of the esophagus using CT may be simple and useful for predicting AGP after AFA. High‐risk patients who have middle‐positioned esophagus or who underwent excessive posterior wall ablation should be followed up closely.

## Graphical abstract

This study revealed that 3.3% of patients following index atrial fibrillation ablation had acute gastroparesis (AGP) using rigorous screening tests after ablation. Middle‐positioned esophagus and posterior wall ablation were risk factors of AGP.

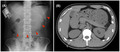

## 1 INTRODUCTION

Atrial fibrillation ablation (AFA) has been the standard non‐pharmacological treatment for atrial fibrillation (AF) since the pulmonary veins (PVs) were proven to be the source of the trigger for AF.^1^ However, this procedure requires ablation of the posterior wall of the left atrium (LA) near the esophagus; therefore, thermal injury can sometimes result in esophageal complications. Atrio‐esophageal fistula is the most devastating but rarest condition.^2^ In contrast, acute gastroparesis (AGP) is relatively common in clinical practice.^3^, ^4^

AGP is a gastric motility disorder that impairs gastric peristalsis by damaging the vagal nerve coursing longitudinally along the esophagus.^3^ Although it is not a life‐threatening disease, it can deteriorate the quality of life, and invasive treatment is required in some cases.^5^ AGP is often asymptomatic and not well recognized by clinicians.^4^ Several clinical studies have reported risk factors for AGP, such as lower body mass index (BMI), small LA, additional ablation in the LA, and insertion of an esophageal probe.^5^, ^6^, ^7^ However, the roles of the anatomical characteristics of the esophagus have not been fully elucidated in the literature.^6^, ^8^, ^9^ In addition, since we have encountered a severe case of AGP requiring long‐term hospitalization, fasting abdominal X‐ray radiography following AFA has been routinely performed for every patient as a screening test for AGP in our hospital.

We aimed to clarify the incidence of AGP using this screening test and to evaluate the risk factors of AGP using pre‐procedural computed tomography (CT).

## 2 METHODS

### 2.1 Study population

This was a retrospective observational study involving 445 patients who underwent initial AFA between November 2015 and December 2020 at our center. This study included persistent AF, defined as AF lasting more than 1 week.^10^ The ablation devices included both conventional radiofrequency catheter ablation (RFA) and cryoballoon ablation (CBA). The exclusion criteria were as follows: (i) contrast‐enhanced CT could not be obtained before AFA (n = 4, chronic kidney disease), (ii) history of esophagogastric disease (n = 15, including gastroesophageal reflux disease, achalasia, esophageal diverticulum, and post‐surgical history of gastric cancer), and (iii) gastroparesis related to other causes (n = 4, including insulin‐dependent diabetes mellitus, amyloidosis, and Parkinson's disease).^11^ The remaining 422 patients were enrolled in the study. Medical charts were reviewed, and clinical data (patients’ background, comorbidities, echocardiographic data, CT data, and the details of ablation) were collected. This study protocol was approved by the ethics committee at our center (approval number: 20C221), and written informed consent was obtained from the patients.

### 2.2 CT protocol

**FIGURE 1:**
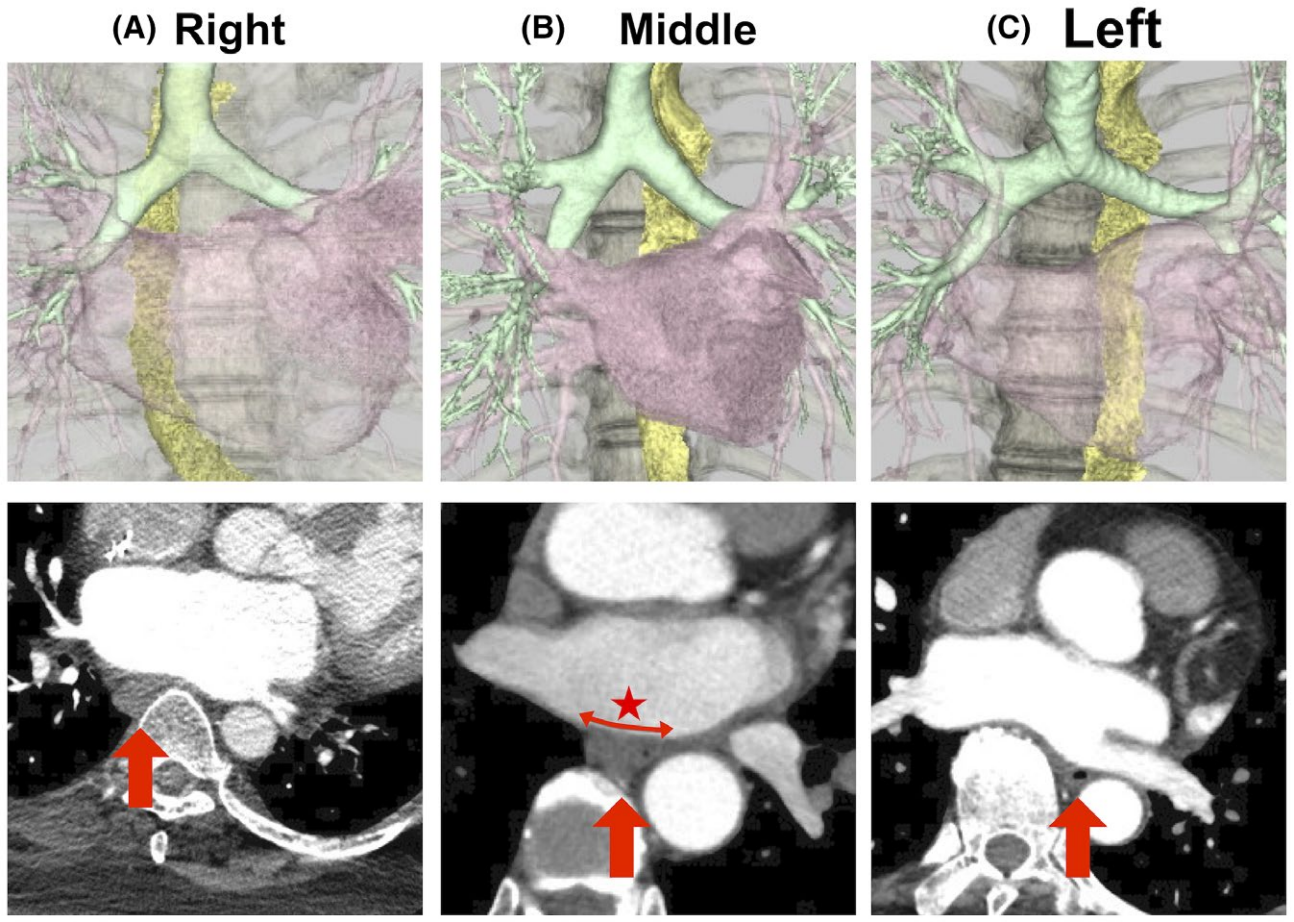
Anatomical classification of the esophagus. The images above are reconstructed using a computed tomography (CT) workstation to visualize the left atrium, esophagus, vertebrae, and trachea. The position of the esophagus was classified to 3 groups (A, right; B, middle; and C, left), regarding the relationship between the esophagus and vertebrae. The images below represent the transverse planes of CT. The red arrow indicates the esophagus. The red star illustrates the transverse width of the esophagus in contact with the posterior wall of the left atrium

All patients underwent contrast‐enhanced CT within 1 month before catheter ablation to confirm the PV anatomy and exclude intracardiac thrombi. We performed 0.5‐mm slice helical‐scanning CT (Aquilion One Vision Edition, Canon Medical Systems) with an intravenous administration of iopamidol (24.5 mg/kg/s). Thereafter, we reconstructed images of the LA and adjacent structures (esophagus, vertebrae, and trachea) using a workstation system (SYNAPSE VINCENT). Using this system, a cardiologist blinded to this study protocol recorded the distance between the esophagus and the LA (LA‐Eso distance) and the transverse width of the esophagus in contact with the posterior wall of the LA at the level of the inferior PVs (Eso width) (Figure 1).^9^ These values were measured twice, and the average value was used for the analysis. In addition, we classified the anatomical location of the esophagus at the level of the PVs into three categories as follows: (i) *left side*, when the longitudinal midline of the esophagus was located over the left edge of the vertebrae; (ii) *middle* (on vertebrae), when the midline of the esophagus was centered on the vertebrae and (iii) *right side*, when the midline of the esophagus was located over the right edge of the vertebrae). Two blinded cardiologists classified the patients into the aforementioned three categories. When their judgments differed, a third blinded cardiologist resolved the discrepancy.

### 2.3 Ablation procedure

Anticoagulation was initiated at least 1 month before the ablation. Antiarrhythmic medications were discontinued at five half‐lives before admission. Oral administration of a proton‐pump inhibitor or potassium‐competitive acid blocker (vonoprazan) was started before AFA and continued until 1 month after ablation. An esophageal probe (Fe‐po ET Watcher, Fukuda Denshi C. Ltd., Tokyo, Japan) was inserted in most cases to detect esophageal temperature changes during AFA. A straight stylet for the esophageal probe was used during insertion, and the stylet was removed after insertion. In some cases, the patients were sedated with intravenous dexmedetomidine and propofol, and the laryngeal mask airway was used to open the airway (deep sedation). Other patients were sedated with dexmedetomidine alone (conscious sedation). Muscle relaxants were not used in both cases. After sedation, sheaths were inserted via the right subclavian vein and bilateral femoral veins. After activated clotting time was adjusted to more than 300 seconds, two long sheaths (8.5‐F SL‐0, Abbott Laboratories, Abbott Park, IL, USA, and 8‐F Preface sheath, Biosense Webster Inc, Diamond‐Bar, CA, USA) were inserted into the LA using the Brockenbrough technique.

For RFA, pulmonary vein isolation (PVI) was performed using a contact‐sensing ablation catheter (Thermocool Smarttouch SF D/D, Biosense Webster Inc) and a three‐dimensional electroanatomical mapping system (CARTO 3 system, Biosense Webster Inc). During ablation at the posterior wall near the esophagus, contact force was limited to 10 g or less. Ablation energy had two patterns as follows: low (less than 25 W) and high output (40 W or greater), using the guidance of the force–time integral (within 100 gs) or the ablation index (within 350). Ablation was stopped when the esophageal temperature was 40°C or higher. Ablation lesions were created while avoiding the longitudinal crossing of the esophagus. Additional linear ablation, such as the roof and floor line (in the case of both linear ablations performed, LA posterior wall isolation), superior vena cava isolation, and cavotricuspid isthmus ablation, was performed at the physician's discretion.

In the case of CBA, a 28‐mm second‐generation cryoballoon catheter (Arctic Front Advance; Medtronic Inc) was used to isolate the PV electrically for 180 seconds per application. The application was discontinued when the distal balloon temperature fell below −60°C or when the esophageal temperature fell below 15°C. If PVI could not be achieved even after three applications, additional ablation (touch‐up ablation) was performed using a radiofrequency ablation catheter (Navistar Thermocool catheter; Biosense Webster Inc).

### 2.4 Diagnosis of AGP after catheter ablation

**FIGURE 2:**
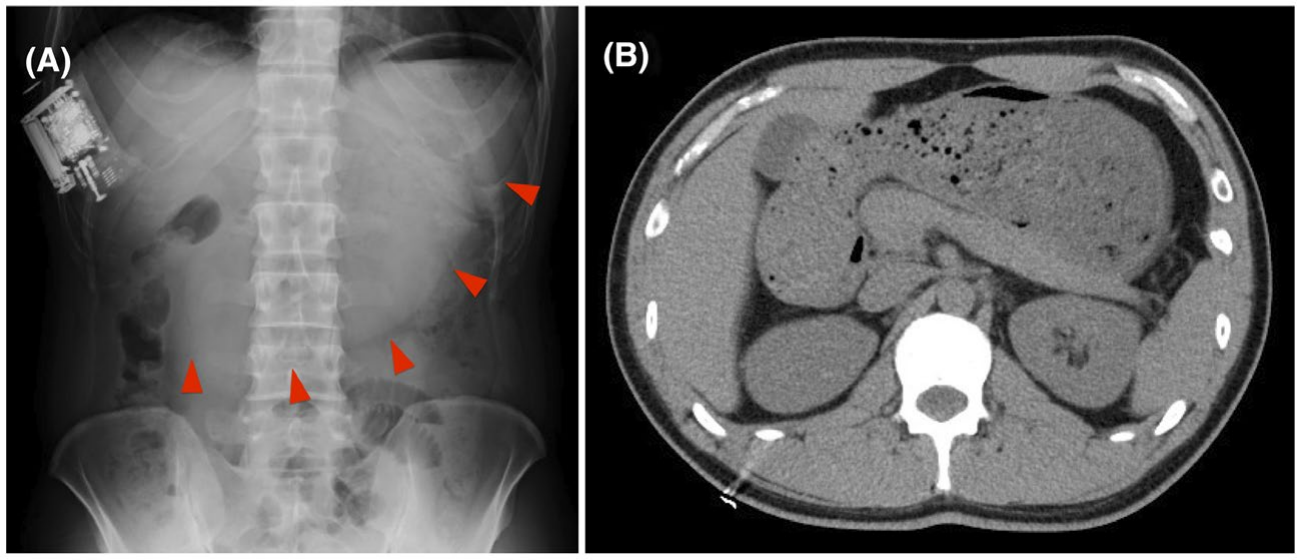
An Abdominal X‐ray radiography was obtained after catheter ablation as the screening test. Red arrows show delayed gastric emptying, therefore this case was diagnosed as acute gastroparesis (AGP) (A). In case that this screening test was inconclusive, the abdominal computed tomography was additionally obtained to diagnose AGP (B)

Symptom assessment and abdominal X‐ray radiography were performed for all patients after AFA. Patients were allowed to have meals on the day after AFA, and subsequently, fasting abdominal X‐ray radiography was performed on the second day at an interval of more than 15 hours after the last meal as the screening test for AGP in all patients. When patients had obvious symptoms related to AGP, and the image screening test was positive, AGP was definitively diagnosed. In other words, AGP was diagnosed when (i) patients were symptomatic and X‐ray screening was positive (Figure 2A), or (ii) patients were symptomatic, X‐ray screening was inconclusive, and additionally performed abdominal CT screening was positive for AGP (Figure 2B). When patients were asymptomatic and X‐ray screening was positive, fasting was continued, and AGP was definitively diagnosed if the imaging test was positive for next two consecutive days. AGP was excluded when patients were asymptomatic, and the imaging test was negative. Patients were classified into two groups according to the occurrence of AGP; thereafter, the differences in clinical and anatomical parameters were compared.

### 2.5 Statistical analysis

Continuous variables are expressed as mean ±standard deviation or median and interquartile range. The clinical and procedural characteristics of the two groups were compared using the Student's *t‐*test or Mann‐Whitney *U* test for continuous variables and Fisher's exact test for categorical variables. Categorical variables are represented as percentages. The kappa (κ) statistic was used to verify inter‐observer agreement in judging the anatomical location of the esophagus, and good agreement was defined as *κ* > 0.6. Logistic regression analysis was used to identify the predictive factors of AGP, and previously reported factors were included in multivariate analysis.^5^, ^6^, ^7^, ^8^, ^9^ These analyses were conducted using JMP^®^ 13 software (SAS Institute Inc).

## 3 RESULTS

### 3.1 Incidence and characteristics of AGP

Clinical characteristics of AGP are shown in Table 1. AGP developed in 14 patients (3.3%) following AFA. Eight patients (1.9% of all patients, 57.1% of patients with AGP) were symptomatic, and the most common symptom was abdominal bloating (7 patients). Six patients (42.9%) were asymptomatic; therefore, a diagnosis was established using serial fasting abdominal X‐ray radiography. AGP resolved in all patients within 4 weeks without invasive treatment.

**TABLE 1:** Clinical characteristics of patients with acute gastroparesis

No.	Age/sex	Eso location	Eso width	LA‐Eso distance	Procedure	Symptom	Timing of recovery
1	42/M	Middle	22.4	2.5	C‐PVI	AB, N/V	4 weeks
2	64/M	Middle	23.7	2.2	C‐PVI+SVCI	none	4 weeks
3	74/M	Left	16.7	3.5	C‐PVI	none	2 weeks
4	41/F	Middle	21.0	2.3	R‐PVI+SVCI	AB	4 weeks
5	52/M	Left	17.7	2.2	R‐PVI+PWI	AB	2 weeks
6	78/F	Middle	22.0	2.2	R‐PVI+Roof	none	1 week
7	72/M	Left	18.4	2.8	R‐PVI+PWI	AB, N/V	4 weeks
8	82/M	Left	17.4	2.4	R‐PVI	none	2 weeks
9	71/F	Left	20.4	1.8	C‐PVI	AB	3 weeks
10	81/F	Middle	17.3	2.1	C‐PVI	none	2 weeks
11	59/M	Left	20.4	2.1	C‐PVI	N/V	1 week
12	68/M	Left	22.0	1.9	R‐PVI+PWI	AP, AB	1 week
13	58/M	Middle	14.4	2.6	R‐PVI+CTI	none	1 week
14	71/M	Left	15.2	2.2	R‐PVI	AB	2 weeks

Abbreviations: AB, abdominal bloating; AP, abdominal pain; C‐PVI, pulmonary vein isolation with cryoballoon; CTI, cavotricuspid isthmus ablation; Eso, esophagus; F, female; LA, left atrium; M, male; N/V, nausea and vomiting; R‐PVI, pulmonary vein isolation with radiofrequency catheter; SVCI, superior vena cava isolation.

### 3.2 Comparisons between the AGP and non‐AGP group

Patient characteristics are shown in Table 2. In all patients, CT before AFA revealed that the esophagus was located on the left side in 85.5%, at the middle position in 12.6%, and on the right side in 1.9%. The inter‐examiner agreement for these three categories of esophageal location was good (*κ* = 0.71; 95% confidence interval [CI], 0.52‐0.88; *P* < .0001).

**TABLE 2:** Patients’ characteristics

	All	AGP	non‐AGP	*P* value
N	422	14	408	
Age—years	67 ± 11	65 ± 13	67 ± 11	.6
Male—n (%)	289 (68.5)	10 (71.4)	279 (68.4)	.8
BMI—kg/m^2^	24.2 ± 3.8	22.7 ± 3.0	24.2 ± 3.8	.2
CHA_2_DS_2_‐Vasc score	2.5 ± 1.7	2.6 ± 2.2	2.5 ± 1.7	.7
Persistent AF—n (%)	184 (43.6)	7 (50.0)	155 (43.4)	.6
EF—%	65.2 ± 10.3	64.1 ± 12.9	65.2 ± 10.2	.7
LAD—mm	40.2 ± 6.9	39.4 ± 10.2	40.2 ± 6.8	.7
PPI—n (%)	182 (43.1)	6 (42.9)	176 (43.1)	1
Vonoprazan—n (%)	240 (56.9)	8 (57.1)	232 (56.9)	
LA‐Eso distance—mm	2.5 ± 0.7	2.3 ± 0.4	2.5 ± 0.7	.4
Eso width—n (%)	19.5 ± 3.5	19.2 ± 2.9	19.5 ± 3.6	.7
Eso (left)—n (%)	361 (85.5)	8 (57.1)	353 (86.5)	.**01**
Eso (middle)—n (%)	53 (12.6)	6 (42.9)	47 (11.5)	
Eso (right)—n (%)	8 (1.9)	0 (0.0)	8 (2.0)	
Deep sedation—n (%)	56 (13.3)	2 (14.3)	54 (13.2)	1
Use of Eso probe—n (%)	388 (91.9)	12 (85.7)	376 (92.2)	.4
RFA—n (%)	153 (36.3)	8 (57.1)	145 (35.5)	.1
CBA—n (%)	269 (63.7)	6 (42.9)	263 (64.5)	

Note

Data are represented as mean ±standard deviation or n (%). Values in bold are significant.

Abbreviations: AF, atrial fibrillation; AGP, acute gastroparesis; BMI, body mass index; CBA, cryoballoon ablation; EF, ejection fraction; Eso, esophagus; LA, left atrium; LAD, left atrium diameter; PPI, proton pump inhibitor; RFA, radiofrequency catheter ablation.

Comparing the patients’ backgrounds (Tables 2 and 3), there were no relationships between the AGP and non‐AGP groups regarding the clinical background. However, the esophagus was observed to be more frequently located at the middle position in the AGP group compared to the non‐AGP group (42.9% vs 11.5%, *P* = .01). Moreover, additional posterior wall ablation was performed more frequently in the AGP group than in the non‐AGP group (50.0% vs 14.5%, *P* = .02).

**TABLE 3:** Procedural characteristics

	All	AGP	non‐AGP	*P* value
RFA				
N	153	8	145	
Low output—n (%)	79 (51.6)	6 (75.0)	73 (50.3)	.2
High output—n (%)	74 (48.4)	2 (25.0)	72 (49.7)	
PW ablation—n (%)	25 (16.3)	4 (50.0)	21 (14.5)	.**02**
Roof line alone—n (%)	3 (2.0)	1 (12.5)	2 (1.4)	
PW isolation—n (%)	22 (14.4)	3 (37.5)	19 (13.1)	
SVCI—n (%)	37 (24.2)	1 (12.5)	36 (24.8)	.4
CTI—n (%)	20 (13.1)	1 (12.5)	19 (13.1)	1.0
CBA				
N	269	6	263	
Freezing time at LSPV—s	240 [180, 360]	360 [270, 398]	220 [180, 360]	.1
Freezing time at LIPV—s	180 [180, 303]	180 [180, 240]	180 [180, 308]	.6
Freezing time at RSPV—s	180 [180, 280]	180 [180, 180]	180 [180, 280]	.3
Freezing time at RIPV—s	310 [180, 360]	180 [180, 510]	310 [180, 360]	1.0
Nadir temp at LSPV—°C	−52.7 ± 4.7	−50.8 ± 4.2	−52.7 ± 4.7	.3
Nadir temp at LIPV—°C	−46.3 ± 10.1	−47.6 ± 2.1	−46.3 ± 10.2	.8
Nadir temp at RSPV—°C	−55.4 ± 5.4	−53.8 ± 3.5	−50.3 ± 7.0	.2
Nadir temp at RIPV—°C	−50.3 ± 6.9	−51.6 ± 5.3	−55.5 ± 5.3	.1
Minimum Eso temp—°C	20.7 ± 9.6	24.1 ± 10.5	20.6 ± 9.5	.4
Touchup ablation—n (%)	49 (18.2)	1 (16.7)	48 (18.3)	.9
SVCI—n (%)	24 (8.9)	1 (16.7)	23 (8.8)	.5
CTI—n (%)	43 (16.0)	0 (0.0)	43 (16.4)	.1

Note

Data are represented as mean ± standard deviation or median and [interquartile range] or n (%). Values in bold are significant.

Abbreviations: AGP, acute gastroparesis; CBA, cryoballoon ablation; CTI, cavotricuspid isthmus ablation; Eso, esophagus; LIPV, left inferior pulmonary vein; LSPV, left superior pulmonary vein; PW, posterior wall; RFA, radiofrequency catheter ablation; RIPV, right inferior pulmonary vein; RSPV, right superior pulmonary vein; SVCI, superior vena cava isolation; temp, temperature.

### 3.3 Predictors of AGP after AFA

Logistic regression analysis (Table 4) revealed that a middle‐positioned esophagus was significantly associated with AGP. Among patients undergoing RFA, posterior wall ablation is equally associated with AGP. The same results were obtained in the multivariate analysis.

**TABLE 4:** Prediction factor of acute gastroparesis

	*P* value	OR (95% of CI)
*Univariate analysis*		
BMI*	.1	0.89 (0.75‐1.03)
LA‐Eso distance*	.4	0.69 (0.27‐1.47)
Eso width*	.7	0.98 (0.83‐1.03)
Middle‐positioned Eso	.**004**	5.76 (1.82‐17.29)
Deep sedation	.9	1.09 (0.17‐4.15)
Usage of Eso probe	.4	0.51 (0.13‐3.37)
PW ablation+	.**006**	7.37 (1.90‐24.16)
CBA	.1	0.41 (0.13‐1.21)
*Multivariate analysis (model 1)*		
BMI*	.1	0.89 (0.76‐1.90)
Middle‐positioned Eso	.**004**	5.96 (1.88‐18.06)
*Multivariate analysis (model 2)*		
LA‐Eso distance*	.5	0.76 (0.30‐1.63)
Middle‐positioned Eso	.**005**	5.53 (1.75‐16.72)
*Multivariate analysis (model 3)*		
Eso width*	.2	0.91 (0.77‐1.06)
Middle‐positioned Eso	.**001**	7.31 (2.17‐23.73)
*Multivariate analysis (model 4)*		
Usage of Eso probe	0.3	0.39 (0.09‐2.66)
Middle‐positioned Eso	.**003**	6.20 (1.94‐19.09)
*Multivariate analysis, among RFA patients (model 5)*		
PW ablation+	.**01**	7.59 (1.53‐42.05)
Middle‐positioned Eso+	.**02**	8.97 (1.51‐53.25)

Note

* indicates per 1 unit OR. + indicates the analysis among RFA patients. Values in bold are significant.

Abbreviations: BMI, body mass index; CBA, cryoballoon ablation; CI, confidence interval; Eso, esophagus; LA, left atrium; OR, odds ratio; PW, posterior wall; RFA, radiofrequency catheter ablation.

## 4 DISCUSSION

The main findings of this study are as follows: (i) The incidence of AGP was 3.3% (symptomatic AGP was 1.9%); (ii) the most common symptom of AGP was abdominal bloating; however, 42.9% of the patients were asymptomatic, and AGP resolved in all patients within 4 weeks without invasive treatment; and (iii) the middle‐positioned esophagus on pre‐procedural CT was an independent anatomical predictor of AGP.

The esophagus is contained and fixed within the mediastinum; however, it is not covered with serosa, unlike other gastrointestinal segments. The efferent vagal nerve courses longitudinally on the anterior wall of the esophagus and connects to the lesser curvature of the stomach through the esophageal hiatus.^12^ The vagal nerve contains motor fibers and is involved in gastric peristalsis. When the vagal nerve is injured by catheter ablation, gastric peristalsis is suppressed, causing delayed gastric emptying. The incidence of AGP after AFA varies from 0.2% to 48% in the literature.^4^, ^5^, ^6^, ^13^, ^14^ According to a study that used empirical testing (esophageal manometry, gastric emptying study, and sham‐feeding test) before and after AFA, 48.1% of patients had delayed gastric emptying after AFA, and most patients were asymptomatic.^4^ These findings are extremely important; AGP is a common but silent disease that can be overlooked as a complication. In our study, 42.9% of patients with AGP were asymptomatic. Generally, the treatment for AGP is conservative; fasting and bowel rest, decompression of the stomach, and administration of prokinetic agents (mosapride, metoclopramide, and erythromycin) are recommended.^15^ However, invasive treatments, such as injecting botulinum toxin A into the pyloric sphincter and surgery (esophagojejunal anastomosis), are required in a few cases.^5^, ^15^

Several studies have reported factors predictive of AGP. Miyazaki et al reported that a lower BMI was the only predictor of AGP in 535 patients who underwent RFA.^6^ In our study, univariate analysis showed that a lower BMI was likely to predict AGP; however, this was not statistically significant (*P* = .1). In addition, it is controversial whether monitoring luminal esophageal temperature can prevent esophageal complications. According to a clinical study involving 3695 patients with RFA, the investigators concluded that esophageal temperature monitoring might reduce AGP.^5^ However, another study revealed that the esophageal probe itself could cause the formation of esophageal ulcers.^16^ They speculated that the esophageal probe may act as an antenna for radiofrequency catheters and cause esophageal injury.^16^, ^17^ Moreover, the insertion of an esophageal probe was itself a risk factor for esophageal injury in CBA.^18^ The anterior wall of the esophagus may be compressed between the esophageal probe and the posterior wall of the LA. We speculate that the periesophageal vagal nerve was likely to be injured when the esophagus was compressed between the LA and the vertebrae. Moreover, additional posterior wall isolation with PVI was reported to be a risk factor for AGP.^19^ To prevent AGP, additional linear lesions near the esophagus should be avoided. Even in CBA cases, Aksu et al reported that a lower temperature in the lower PVs during CBA was a risk factor for AGP.^7^ Our results were not consistent with this report; however, the freezing time should be shortened in the lower PVs.

Regarding the anatomical evaluation of the esophagus, predicting complications based on the distance between the LA and the esophagus is controversial.^6^, ^8^ Tsuboi et al reported that esophageal width contacting the LA was greater in patients with vagal nerve injury (VNI) including AGP (VNI vs. non‐VNI, 26.5 ± 6.9 mm vs 19.1 ± 4.9 mm; *P* = .01).^9^ In our study, the esophagus width was similarly observed to be greater in the middle‐positioned than in the left‐sided esophagus (middle vs. left, 21.8 ± 3.8 mm vs 19.1 ± 3.3; *P* < .001). Therefore, the middle‐positioned esophagus may be flattened for anatomical reasons, which may lead to VNI by catheter ablation. Hasegawa et al reported that AGP after PVI with cryoballoon frequently occurred in cases where the esophagus was located between the left and right inferior PV ostia, which is equivalent to the middle‐positioned esophagus in our study. This result is consistent with ours.^20^ Moreover, esophageal contraction can be observed during catheter ablation.^21^, ^22^ We speculate that this phenomenon may not occur sufficiently in the case of a middle‐sided esophagus. Nevertheless, further clinical studies using contrast esophagography during AFA are necessary to verify this hypothesis.

This study has several limitations. First, this was a single‐center, retrospective observational study with a relatively small study population. Second, the actual distance between the ablation points and the esophagus could not be measured. Third, data on esophageal temperature were not available for RFA. Finally, endoscopy after AFA was not performed to evaluate direct esophageal injury. To resolve these limitations, a multi‐center, prospective study with a more thorough study protocol, using methods, such as contrast esophagography and endoscopy, is needed.

## 5 CONCLUSION

Our study suggests that AGP is not a rare complication, especially when asymptomatic patients were included. Evaluation of the esophageal anatomy using CT may be simple and useful in predicting the risk of AGP. Although routine fasting abdominal X‐ray radiography may be unnecessary in clinical practice, when the anatomy of the esophagus or ablation procedure suggests that the patient is at high risk, he or she should be followed up closely after catheter ablation.

## CONFLICT OF INTEREST

Authors declare no conflicts of interests for this article. The protocol for this study was approved by the Ethics Committee of our center on February 25, 2021 (Approval number: 20C221).
